# Brief exposure of skin to near-infrared laser augments early vaccine responses

**DOI:** 10.1515/nanoph-2021-0133

**Published:** 2021-08-09

**Authors:** Shinya Yokomizo, Wataru Katagiri, Yohei Maki, Tomoya Sano, Kazumasa Inoue, Masahiro Fukushi, Dmitriy N. Atochin, Toshihiro Kushibiki, Akihiko Kawana, Yoshifumi Kimizuka, Satoshi Kashiwagi

**Affiliations:** Gordon Center for Medical Imaging, Department of Radiology, Massachusetts General Hospital, 149 13th Street, Charlestown 02129, MA, USA; Department of Radiological Sciences, Tokyo Metropolitan University, 7-2-10 Higashi-Ogu, Arakawa 116-8551, Tokyo, Japan; Gordon Center for Medical Imaging, Department of Radiology, Massachusetts General Hospital, 149 13th Street, Charlestown 02129, MA, USA; Graduate School of Science and Technology, Keio University, 3-14-1 Hiyoshi, Yokohama 223-8522, Kanagawa, Japan; Division of Infectious Diseases and Respiratory Medicine, Department of Medicine, National Defense Medical College, 3-2 Namiki, Tokorozawa, Saitama 359-8513, Japan; Division of Infectious Diseases and Respiratory Medicine, Department of Medicine, National Defense Medical College, 3-2 Namiki, Tokorozawa, Saitama 359-8513, Japan; Department of Radiological Sciences, Tokyo Metropolitan University, 7-2-10 Higashi-Ogu, Arakawa 116-8551, Tokyo, Japan; Department of Radiological Sciences, Tokyo Metropolitan University, 7-2-10 Higashi-Ogu, Arakawa 116-8551, Tokyo, Japan; Cardiovascular Research Center, Department of Medicine, Massachusetts General Hospital, 149 13th Street, Charlestown 02129, MA, USA; Department of Medical Engineering, National Defense Medical College, 3-2 Namiki, Tokorozawa, Saitama 359-8513, Japan; Division of Infectious Diseases and Respiratory Medicine, Department of Medicine, National Defense Medical College, 3-2 Namiki, Tokorozawa, Saitama 359-8513, Japan; Division of Infectious Diseases and Respiratory Medicine, Department of Medicine, National Defense Medical College, 3-2 Namiki, Tokorozawa, Saitama 359-8513, Japan; Gordon Center for Medical Imaging, Department of Radiology, Massachusetts General Hospital, 149 13th Street, Charlestown 02129, MA, USA

**Keywords:** adjuvant, CD103^+^ dendritic cells, early antibody response, near-infrared laser, vaccine

## Abstract

Rapid establishment of herd immunity with vaccination is effective to combat emerging infectious diseases. Although the incorporation of adjuvant and intradermal (ID) injection could augment early responses to the vaccine, the current chemical or biological adjuvants are inappropriate for this purpose with their side effects and high reactogenicity in the skin. Recently, a near-infrared (NIR) laser has been shown to augment the immune response to ID vaccination and could be alternatively used for mass vaccination programs. Here, we determined the effect of NIR laser as well as licensed chemical adjuvants on the immunogenicity 1, 2, and 4 weeks after ID influenza vaccination in mice. The NIR laser adjuvant augmented early antibody responses, while the widely used alum adjuvant induced significantly delayed responses. In addition, the oil-in-water and alum adjuvants, but not the NIR laser, elicited escalated T_H_2 responses with allergenic immunoglobulin E (IgE) responses. The effect of the NIR laser was significantly suppressed in the basic leucine zipper transcription factor ATF-like 3 (Batf3) knockout mice, suggesting a critical role of the cluster of differentiation 103^+^ (CD103)^+^ dendritic cells. The current preliminary study suggests that NIR laser adjuvant is an alternative strategy to chemical and biological agents to timely combat emerging infectious diseases. Moreover, its immunomodulatory property could be used to enhance the efficacy of immunotherapy for allergy and cancer.

## 1 Introduction

The establishment of herd immunity with vaccination is an effective medical measure to combat the threat of emerging infectious diseases [1]. Rapid design and production of an effective vaccine is, therefore, a major focus of research and development in this field [2–4]. In response to this, a wide array of platform technologies, which is based on DNA, RNA, viral vectors, or bacterial constructs, is in an active development phase for accelerated vaccine development [3, 5–8]. However, even upon timely design, successful production, and rapid distribution with accelerated regulatory approval of vaccines, it often takes weeks or longer for the vaccination and booster as appropriate to confer protection [9] because this time period is required for production and maturation of neutralizing antibody [10, 11].

Since the skin is the frontline of defense, the skin-based immune system is expected to be effectively primed and readied to respond to pathogens [12]. The skin-based vaccination has been tested to generate faster and stronger immune responses than other standard routes of vaccine administration [13–18]. Unfortunately, protection induced by this strategy is still equivalent to the conventional approaches [19], suggesting that simple improvements in vaccine delivery are not sufficient to achieve clinical significance.

The incorporation of adjuvants into vaccine formulations is an established strategy to enhance the immunogenicity of vaccines [20]. Amongst potential benefits of adjuvants, induction of faster antibody response [21–23] holds significant importance to mitigate mortality and morbidity by emerging infectious diseases. This benefit is especially critical for the elderly population with immune senescence and a delayed immune response to vaccination compared to young adults [24–26]. The current adjuvants, however, are linked to undesirable side effects including local reactogenicity and systemic toxicity and only a limited number of adjuvants has been incorporated into clinically licensed vaccines [27, 28]. In addition, these conventional adjuvants are inappropriate for use in the skin with their potential to stimulate strong innate responses [29, 30]. Together, these issues keep preventing intradermal (ID) vaccination with adjuvant from achieving these goals and clinical translation. The development of a new class of adjuvant that facilitates a rapid response with acceptable safety is, therefore, a significant priority for the development of the next generation of vaccine programs.

In the past decade, researchers have consistently reported that skin treatment with near-infrared (NIR) laser activates innate immunity and augments the immune response to the vaccine [31–33]. Currently, four classes of laser adjuvants have been established; ultra-short pulsed [34], nonpulsed [34–38], nonablative fractional [39–43], and ablative fractional [44–52] lasers. Each laser adjuvant shows a distinct mode of action, but all of them have been shown to stimulate innate responses and show adjuvant effects on vaccines.

Amongst these laser adjuvants, nontissue damaging, continuous wave (CW) NIR laser holds a promise in clinical translation. Brief exposures of skin with nontissue damaging NIR laser have been reported to augment ID vaccination without applicable side effect [34, 35] and activate innate responses of the cluster of differentiation 103^+^ (CD103^+^) migratory dendritic cells (migDCs) in the skin [36, 37], which are pivotal in early and long-term adaptive memory responses [53–57]. The X-C motif chemokine receptor 1 (XCR1^+^) dendritic cell (DC) subset is increasingly recognized as being critical for the formation of cytotoxic T cell and T helper cell type 1 (T_H_1) responses, long-term adaptive memory response [53–57], and early and long-term antibody response [58]. XCR1^+^ DCs largely correspond to lymph node-resident CD8α^+^ and CD103^+^ migDCs [57, 59]. These reports together suggest that the NIR laser could augment early antibody responses upon vaccination via activation of CD103^+^ migDCs, but the potential of the laser adjuvant to accelerate such responses is not determined to date.

In this preliminary study, through comparison with the conventional chemical adjuvants, we found that the laser adjuvant augmented early antibody responses to ID vaccination with acceptable safety.

## 2 Materials and methods

### 2.1 Animals

Eight-week-old female C57BL/6J (stock no: 000664) and breeding pairs of the basic leucine zipper transcription factor ATF-like 3 knockout (Batf3^−/−^)mice (013755) were purchased from Jackson Laboratories (Bar Harbor,ME). All animals were acclimated for at least two weeks prior to use for the experiments. Batf3^−/−^ mice were bred at Massachusetts General Hospital (MGH). We performed all animal procedures following the Public Health Service Policy on Humane Care of Laboratory Animals. All animal procedures were reviewed and approved by the Institutional Animal Care and Use Committee of MGH (2009N000103).

### 2.2 The design and assembly of a near-infrared (NIR) laser device

CW neodymium-doped yttrium aluminum garnet (Nd:YAG) laser (λ = 1064 nm, ventus) from Laser Quantum (Stockport, Cheshire, UK) was used for a laser source throughout the study. The 1064 nm laser beam was directed to multimode optic fiber (Core: 200 μm, NA: 0.22, Thorlabs, USA) by an achromatic lens (AC127-025-C, Thorlabs). The diverging laser was collimated by a plano-convex lens (LA1074-C, Thorlabs, USA). To obtain a homogenized flat-top intensity distribution of the beam, a holographic diffuser (47-680, Edmund Optics, USA) was used in the optical path. The beam diameter was then set to be 5 mm by adjusting the diameter of an aperture (Thorlabs) and the distance between the iris and the irradiation plane of an animal. The laser device produced a circular target of 0.2 cm^2^ in size, which was confirmed by the infrared images of the spot as described previously [35].

### 2.3 Application of NIR laser adjuvant

**Figure 1:**
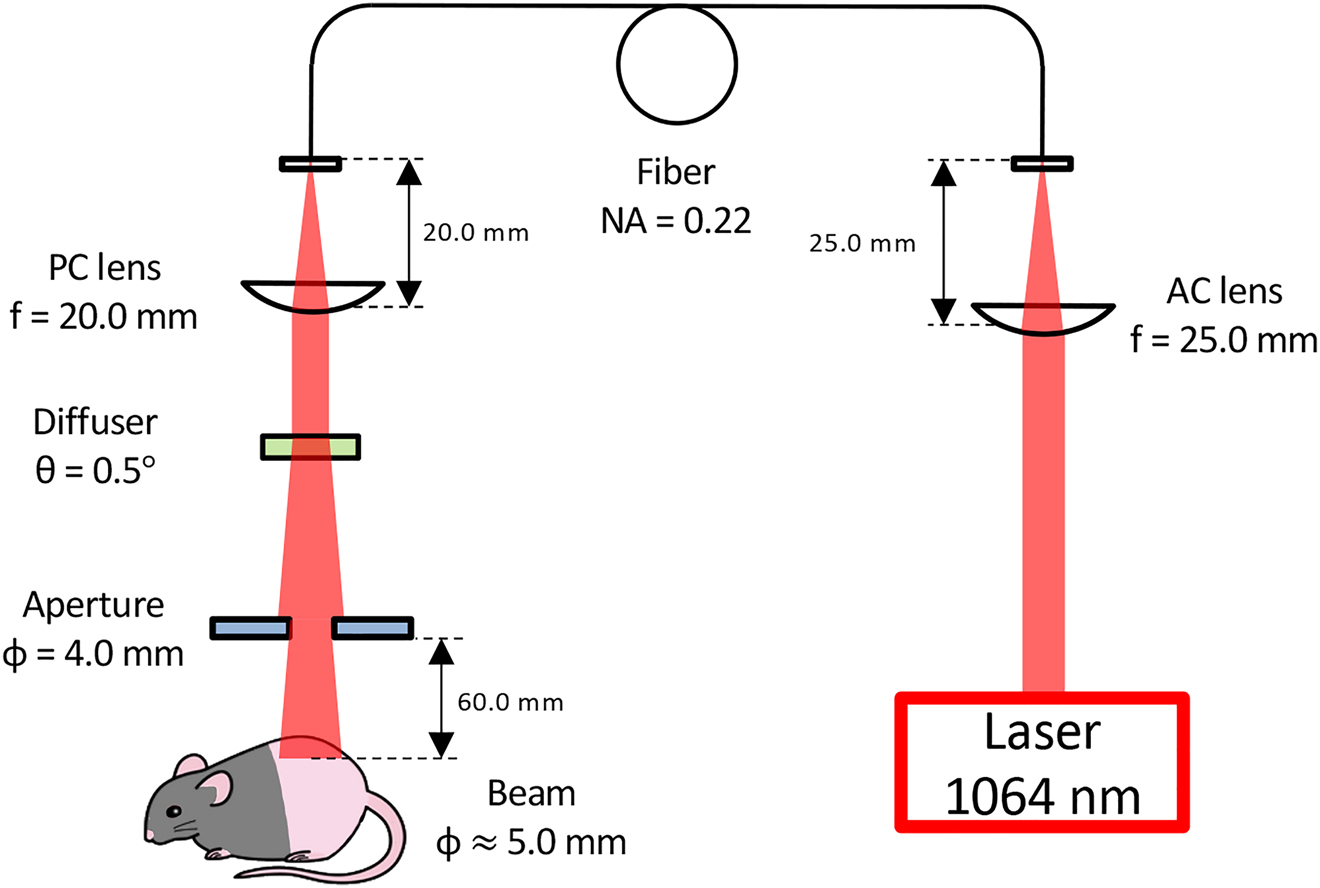
A schematic of the laser irradiation system.A continuous wave (CW) Nd:YAG laser (λ = 1064 nm) was used as a source of near-infrared (NIR) light. The 1064 nm beam was directed to multimode optic fiber (Core: 200 μm, NA: 0.22) by an achromatic lens. To disperse the beam mode and obtain a homogenized flat-top intensity distribution, a holographic diffuser was used in the optical path. The diverging laser was collimated by a plano-convex lens. The beam diameter was set to be 5 mm by adjusting the diameter of an aperture and the distance between the iris and an animal. NA: numerical aperture, AC: achromatic, PC: plano-convex.

Two days before the laser treatment, mice were depilated using a commercial hair remover (Nair, Church, and Dwight). An established nontissue damaging irradiance of 5 W/cm^2^ was used for this study as previously described [34, 36, 37]. We adjusted the irradiance using a power meter for each illumination (Thorlabs) so that the irradiance on a circular spot at the skin surface measured 5 W/cm^2^. The CW 1064 nm Nd:YAG laser was applied on a circular spot of 5 mm in diameter on the skin surface for 1 min on the shaved back skin on a custom-made stage (Figure 1). The skin temperature was monitored throughout the laser application using an infrared thermal imager (FLIR Systems, North Billerica, MA) as previously described [34, 35].

### 2.4 Influenza vaccination models in mice

We examined the efficacy of the laser adjuvant in an established mouse model of influenza vaccination [34, 36, 37]. Immediately after the completion of the application of the laser adjuvant on the back skin of mice, the whole inactivated influenza virus A/PR/8/34 (H1N1) (1 μg, Charles River) in 10 μL saline was intradermally injected in the center of the laser-treated spot. The vaccine mixed with alum (diluted 1 : 1 v/v, Imject, Thermo-Fisher) or an oil-in-water emulsion adjuvant with a formulation similar to MF59 (diluted 1 : 1 v/v, AddaVax, InvivoGen) was used for comparison as appropriate. Blood samples for further analysis were taken 1, 2, and 4 weeks after the vaccination. For visual inspection of skin damage, we observed for any signs of skin damage including blistering, bruising, crusting, edema, redness, swelling, or hair loss during and at 1 and 2 weeks after laser and chemical adjuvant applications as previously described [34, 35] and took photographs of the back skin.

### 2.5 Anti-influenza antibody titers

We determined anti-influenza-specific immunoglobulin G (IgG), IgG1, IgG2c, and immunoglobulin E (IgE) responses by enzyme-linked immunosorbent assay (ELISA) as previously described [34]. Briefly, Immulon plates (Thermo Scientific) were coated with 100 ng/well of the inactivated influenza virus overnight, and serially diluted serum samples were then added to the wells after the plates were blocked. Bound immunoglobulins were detected with the secondary antibody (goat anti-mouse IgG [1 : 10,000, Sigma-Aldrich], rat anti-mouse IgG1 [1 : 2000, SouthernBiotech], goat anti-mouse IgG2c [1 : 4000, SouthernBiotech], or rat antibody to mouse IgE [1 : 1000, SouthernBiotech]) as appropriate. In the case of IgE, the wells were coated with 1 μg/well of the inactivated virus, and the plate was further treated with ELAST ELISA Amplification System (PerkinElmer) after the application of the secondary antibody to improve the sensitivity of the assay. At the end of the incubation, we added tetramethylbenzidine (TMB) substrate (1-Step Ultra TMB, Thermo-Fisher) to the wells. We measured the absorption at 450 nm using an ELISA reader (The SpectraMax iD5 reader, Molecular Devices). For titers of IgG and its subclasses to influenza, a titer was designated as a serum dilution corresponding to an inflection point. For IgE titers to influenza, a statistically defined endpoint antibody titer was determined with a confidence level of 99% [34].

### 2.6 Statistical analyses

For the analysis of serum antibody response, a log transformation of antibody titers was applied in order to reduce positive skewing in the distribution of the raw antibody titers which would violate parametric test assumptions. We ran a two-way analysis of variance (ANOVA) across treatments and time with Tukey adjusted post hoc pairwise mean comparison tests for all statistical analyses unless otherwise specified. The data analysis for this paper was conducted using Prism 8.0.1 (GraphPad software 2018).

## 3 Results

### 3.1 The continuous wave (CW) near-infrared (NIR) laser augments early antibody response

**Figure 2:**
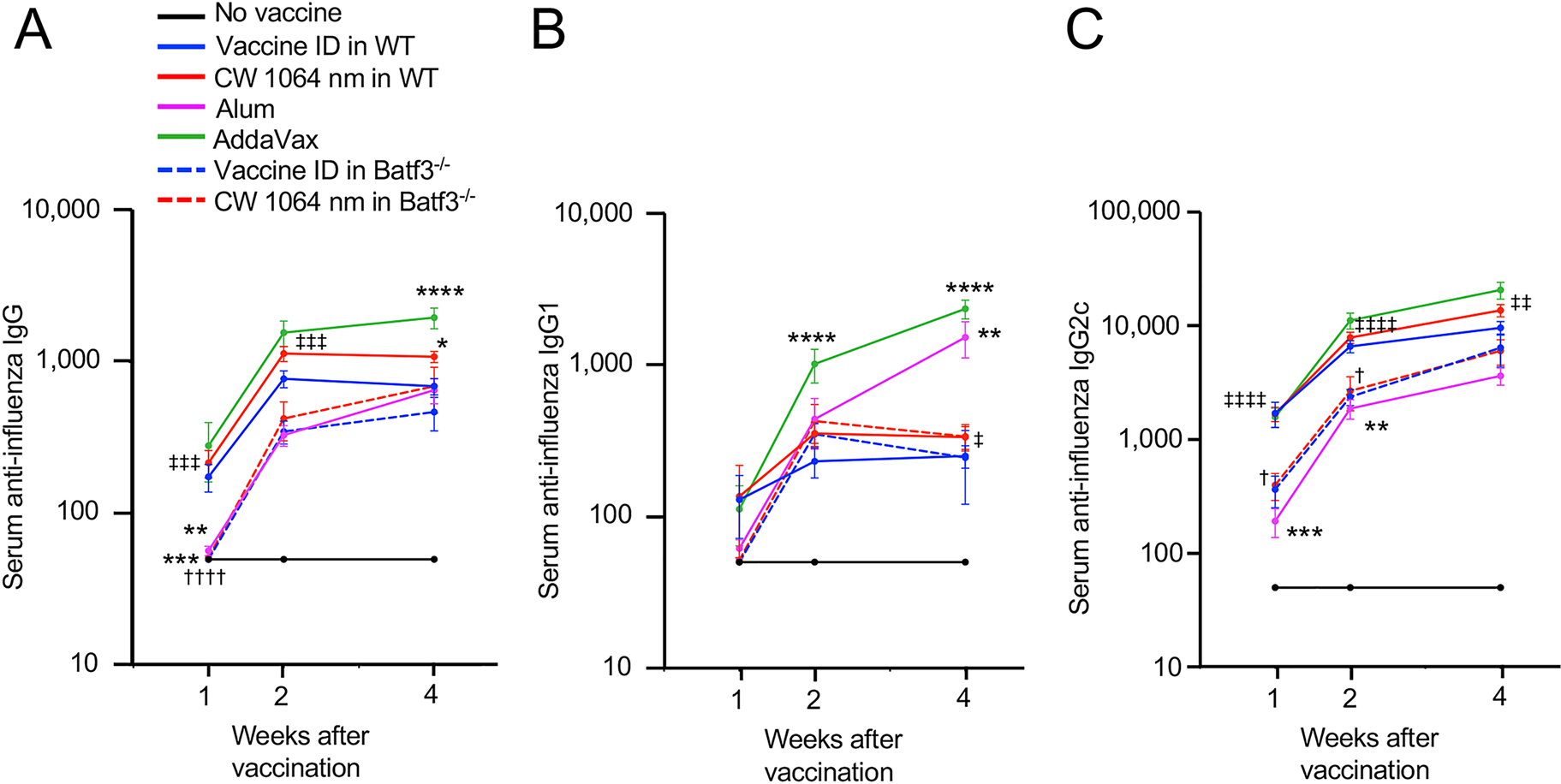
The effect of NIR laser adjuvant on early antibody responses and a critical role of skin the cluster of differentiation 103^+^ (CD103^+^) dendritic cells (DCs) in the antibody responses. The effect of NIR laser on anti-influenza antibody responses was evaluated in wild type (WT) and CD103^+^ DC-deficient the basic leucine zipper transcription factor ATF-like 3 knockout (Batf3^−/−^) mice. WT and Batf3^−/−^ mice were vaccinated intradermally (ID) with 1 μg of inactivated influenza virus (A/PR/8/34) with or without the NIR laser exposure or alum or AddaVax adjuvant. Immune correlates were analyzed at weeks 1, 2, and 4. Plates for enzyme-linked immunosorbent assay (ELISA) were coated with the inactivated influenza virus. Serum anti-influenza specific (A) immunoglobulin G (IgG), (B) IgG1, and (C) IgG2c are shown. (A–C) *n* = 30, 38, 34, 14, 25, 5, 8 for no vaccine, vaccine ID only in WT, vaccine ID + NIR laser in WT, vaccine + Alum ID, vaccine + AddaVax ID, vaccine ID only in Batf3^−/−^, vaccine ID + NIR laser in Batf3^−/−^ groups, respectively. *P* values are based on a two-way treatment by time ANOVA with Tukey post hoc tests. *P < 0.05, ^**^P <0.01, ^***^P < 0.001, ^****^P < 0.0001 as compared to the ID-only group in WT mice; ^†^*P* < 0.05, ^††††^*P* < 0.0001 as compared to the NIR laser group in WT mice; ^‡^*P* < 0.05, ^‡‡^*P* < 0.01, ^‡‡‡^*P* < 0.001, ^‡‡‡‡^*P* < 0.0001 as compared to the alum group.

In order to determine the effect of NIR laser adjuvant and representative licensed chemical adjuvants on early antibody responses, we measured time course kinetics of serum IgG, its subclasses, and IgE titers in an established mouse model of influenza vaccination [34, 36, 37] 1, 2, and 4 weeks after vaccination. Mice were immunized ID with whole inactivated influenza A/PR/8/34 virus with or without CW 1064 nm NIR laser treatment at 5 W/cm^2^ for 1 min [34–36] or representative licensed chemical adjuvant alum or AddaVax (equivalent to MF59). Consistent with the previous reports [34–36], the NIR laser adjuvant increased anti-influenza IgG response at weeks 1 and 2 compared to the ID-only group, and significantly augmented it at week 4 (Figure 2A, the NIR laser vs. ID-only group for IgG: *P* = 0.0125). It similarly increased IgG1 and IgG2c at weeks 2 and 4 compared to the ID-only group (Figure 2B and C).

The AddaVax adjuvant similarly increased IgG titers at weeks 1 and 2, and significantly augmented them at week 4 (Figure 2A, the AddaVax vs. the ID-only group for IgG: *P* < 0.0001). It also increased IgG2c at weeks 2 and 4 to the NIR laser (Figure 2C), and notably, it significantly escalated IgG1 responses at weeks 2 and 4 (Figure 2B, the AddaVax vs. the ID-only group: *P* < 0.0001 for both weeks 2 and 4).

While there is no significant difference in IgG titers between the alum and NIR laser groups at week 4, a level of IgG titers in the NIR laser group is significantly higher than that of the alum group at weeks 1 and 2 (Figure 2A, the NIR laser vs. the alum group: *P* = 0.0005 at week 1 and *P* = 0.0008 at week 2), suggesting that the effect of alum adjuvant shows slower kinetics than that of the NIR laser. Alum adjuvant showed a significant effect on a level of IgG1 titers at week 4 (Figure 2B, the alum vs. the ID-only group: *P* = 0.0016, the alum vs. the NIR laser group: *P* = 0.0110), while a level of IgG1 titers of the alum group was lower at 1 week compared to those of the ID only and NIR laser groups (Figure 2B). In addition, a level of IgG2c titers in the alum group was significantly lower than that of the ID-only group at weeks 1 and 2 (Figure 2C, the ID only vs. the alum group: *P* = 0.0003 at week 1 and *P* = 0.0052 at week 2) and that of the NIR laser group at all the time points (Figure 2C, the NIR laser vs. the alum group: *P* < 0.0001 at weeks 1 and 2 and *P* = 0.0076 at week 4). These results together support the view that alum not only shows slow kinetics but also induces strong T_H_2 responses, which is consistent with the previous reports [60–62].

### 3.2 A critical role of CD103^+^ DCs in the skin in early antibody responses

CD103^+^ migDCs, which are critical in early and long-term adaptive responses [53–58], have been also shown to mediate the adjuvant effect of NIR laser in our previous study [37]. Thus, we further evaluated the contribution of CD103^+^ migDCs to the adjuvant effect using Batf3 deficient (Batf3^−/−^) mice, which lack the cross-presenting CD103^+^ DC subset in the skin [63–65]. In particular, we determined the alterations of the immune response to ID vaccination in the mouse model of influenza vaccination using this genetic mouse model. Consistently with the previous report [37], little adjuvant effect of the NIR laser was observed in Batf3^−/−^ mice in IgG titers and its subclasses at all-time points (Figure 2A–C). Interestingly, a level of IgG titers in the ID only and NIR laser groups in Batf3^−/−^ mice was significantly lower than that in wild type (WT) mice at 1 week (Figure 2A, the ID-only groups in WT vs. Batf3^−/−^ mice: *P* = 0.0005, the NIR laser groups in WT vs. Batf3^−/−^ mice: *P* < 0.0001) and a level of IgG2c titers in the NIR laser group in Batf3^−/−^ mice was significantly lower than that in WT mice at weeks 1 and 2 (Figure 2C, *P* = 0.0351 at week 1, *P* = 0.0461 at week 2), while a comparable level of the antibody responses of the ID only and CW NIR laser groups in Batf3^−/−^ mice to that of the ID-only group in WT mice was observed at week 4 (Figure 2A–C), indicating that Batf3^−/−^ mice show significantly delayed responses and that CD103^+^ DCs play a critical role in early antibody response to ID vaccination.

**Figure 3:**
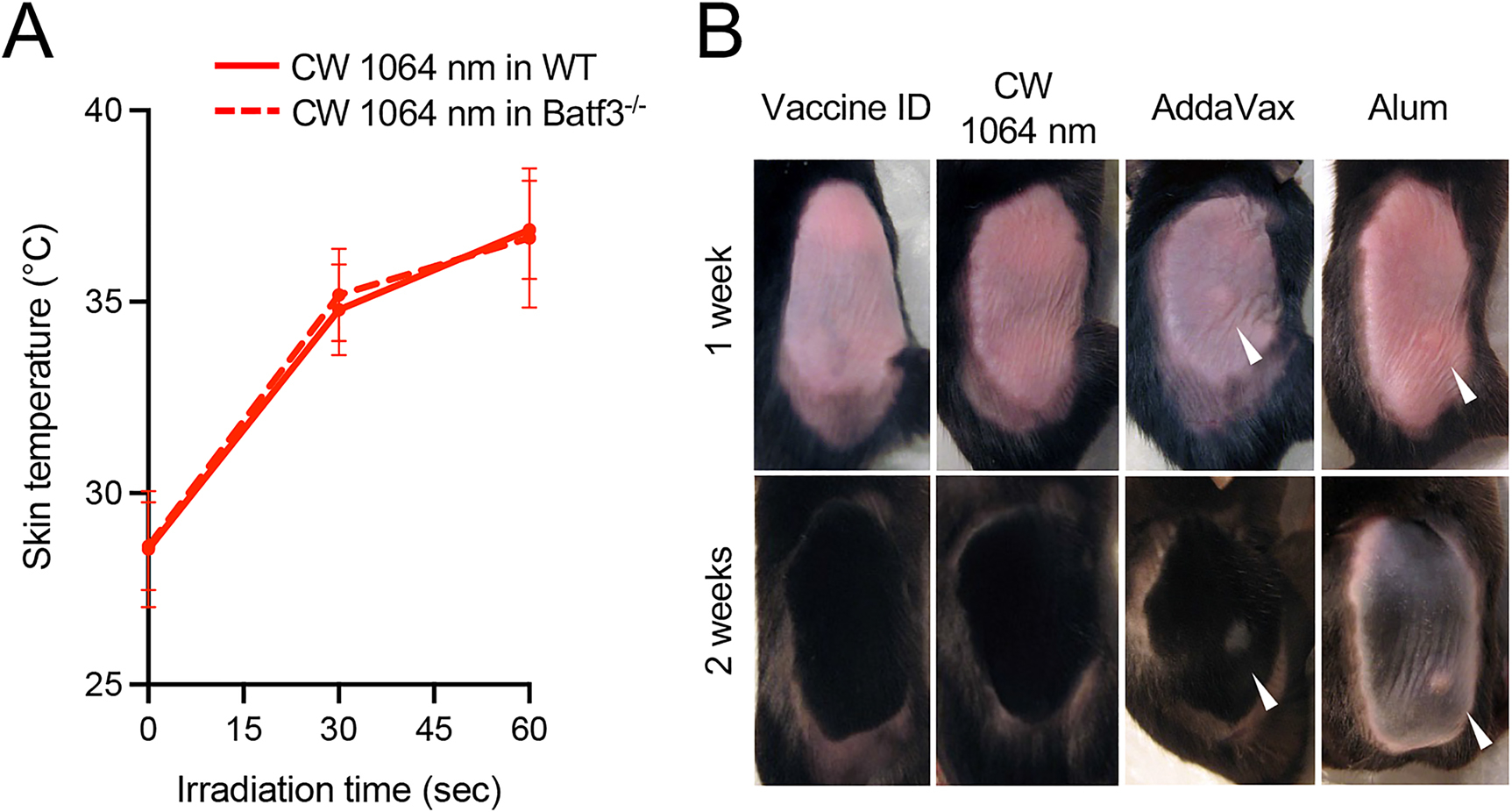
Effect of NIR laser and chemical adjuvants on skin tissue. (A) Dose–temperature responses of the CW 1064 nm laser in mouse skin. Error bars show means ± s.d. *n* = 20, 8 for vaccine ID + NIR laser in WT and vaccine ID + NIR laser in Batf3^−/−^ groups, respectively. (B) Images of the back of mice for visual inspection at 1 and 2 weeks after the application of NIR laser or chemical adjuvant. WT and Batf3^−/−^ mice were vaccinated ID with 1 μg of inactivated influenza virus (A/PR/8/34) with or without the NIR laser exposure or alum or AddaVax adjuvant. *n* = 6, 7, 3, 5 for vaccine ID only in WT, vaccine ID + NIR laser in WT, vaccine + Alum ID in WT, vaccine + AddaVax ID in WT groups, respectively. Representative images for each group are presented.

We have previously identified the maximum nontissue damaging dosage for NIR laser adjuvant. The nontissue damaging dose for 1064 nm NIR laser was determined to be one at which skin temperatures did not exceed 43 °C and for which no visible or microscopic skin damage was detected [34, 35]. Consistently, the skin temperature in WT mice as well as Batf3^−/−^ mice did not exceed 40 °C 1 min after laser irradiation (Figure 3A), suggesting the genetic alteration did not affect the photothermal process in the skin. At this dose, no visible skin damage at any given time point was detected in the laser-treated group (Figure 3B). Contrary, visible skin swelling 1 week and selective hair loss in the injection site 2 weeks after vaccination was observed in the chemical adjuvant groups (Figure 3B), which is consistent with the literature [30]. Thus, we concluded that the dosage of the NIR laser is safe and nontissue damaging, while chemical adjuvant may not be tolerated for ID use.

### 3.3 The effect of NIR laser and chemical adjuvants on an allergenic IgE response

Since alum and AddaVax adjuvant both showed an escalated IgG1 response, we then determined the qualitative changes in the immune response to the NIR laser and chemical adjuvants in the context of ID vaccination.

**Figure 4:**
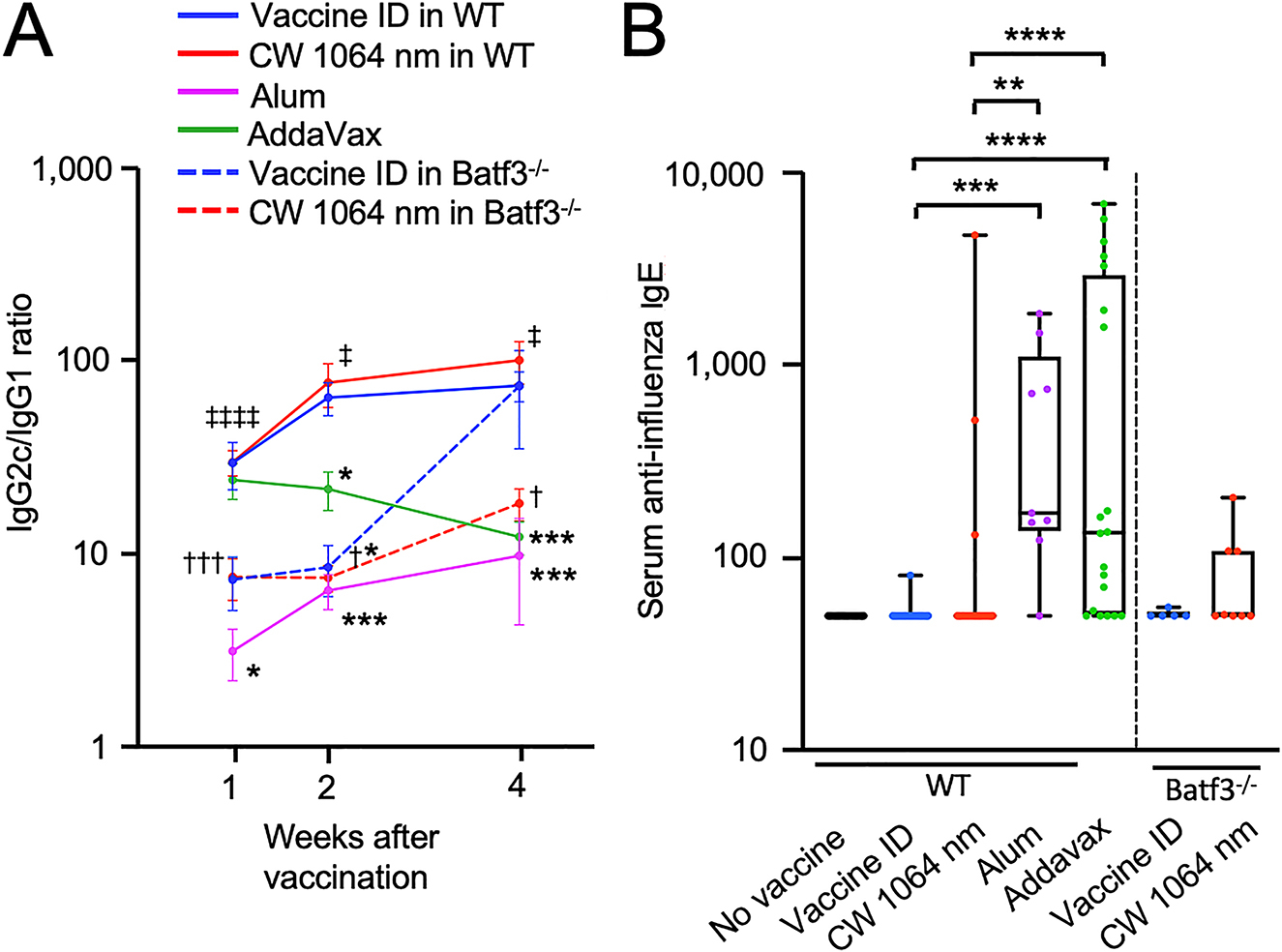
The effect of NIR laser and chemical adjuvants on IgE response. The qualitative changes in the immune response to the NIR laser and chemical adjuvants in the context of intradermal influenza vaccination were assessed in WT and Batf3^−/−^ mice. Serum anti-influenza specific (A) IgG2c/IgG1 ratio, (B) IgE are shown. (A) *n* = 30, 38, 34, 14, 25, 5, 8, (B) *n* = 25, 33, 30, 9, 20, 5, 8 for no vaccine, vaccine ID only in WT, vaccine ID + NIR laser in WT, vaccine + Alum ID, vaccine + AddaVax ID, vaccine ID only in Batf3^−/−^, vaccine ID + NIR laser in Batf3^−/−^ groups, respectively. (A) *P* values are based on a two-way treatment by time ANOVA with Tukey post hoc tests. *P < 0.05, ^***^P < 0.001 as compared to the ID-only group in WT mice; ^†^*P* < 0.05, ^†††^*P* < 0.001 as compared to the NIR laser group in WT mice; ^‡^*P* < 0.05, ^‡‡‡‡^*P* < 0.0001 as compared to the alum group. (B) *P* values are based on a one-way ANOVA with Tukey post hoc tests. ^**^P < 0.01, ^***^P < 0.001, ^****^P < 0.0001.

To assess the T_H_1–T_H_2 balance, we determined IgG2c : IgG1 ratios of the antibody responses in the influenza vaccination model. The ratios of the NIR laser adjuvant group were similar to those of the ID-only group at all the time points (Figure 4A), suggesting that the NIR laser adjuvant does not skew antibody responses to ID vaccination. The ratios of the alum adjuvant group were significantly lower than those of the ID-only group (Figure 4A, *P* = 0.0287 at week 1, *P* = 0.0006 at week 2, and *P* = 0.0005 at week 4) and also lower than those of the NIR laser group (Figure 4A, *P* < 0.0001 at week 1, *P* = 0.0135 at week 2, and *P* = 0.0128 at week 4), indicating T_H_2-skewed responses with the alum adjuvant. Interestingly, the ratios of the AddaVax group were significantly lower than those of the ID-only group at weeks 2 and 4, although the ratios were not significantly different at week 1 (Figure 4A, *P* = 0.0290 at week 2 and *P* = 0.0005 at week 4), indicating that the AddaVax adjuvant progressively skews the response toward T_H_2 in the context of ID vaccination.

The ratios of the ID only and NIR laser groups in Batf3^−/−^ mice were lower than those of the ID-only group in WT mice (Figure 4A), which is consistent with the previous reports showing that the CD103^+^ DC subset is critical for T_H_1 immunity [64]. The ratios of the NIR laser group in Batf3^−/−^ mice were significantly lower than those of the NIR laser group in WTmice at all the time points (Figure 4A, *P* = 0.0006 at week 1, *P* = 0.0154 at week 2, and *P* = 0.0275 at week 4), and the ratios of the ID-only group in Batf3^−/−^ mice were significantly lower than those of the ID-only group in WT mice at week 2 (Figure 4A, *P*=0.0011).

It has been recognized that alum adjuvant induces strong T_H_2 and IgE responses [66, 67]. Consistent with the previous work [34, 35], the alum adjuvant significantly augmented an IgE response to the ID vaccination, while the NIR laser adjuvant did not induce appreciable IgE responses (Figure 4B, the alum vs. the ID-only group: *P* = 0.0001, the alum vs. the NIR laser group: *P* = 0.0017). Similar to the alum adjuvant, the AddaVax adjuvant escalated T_H_2 responses, resulting in augmentation of IgE responses (Figure 4B, the AddaVax vs. the ID-only group: *P* < 0.0001, the AddaVax vs. the NIR laser group: *P* < 0.0001). No appreciable IgE response was observed in any group in Batf3^−/−^ mice (Figure 4B). Together, these results suggest that the ID application of the chemical adjuvants produces a T_H_2-skewed response and induces an allergenic IgE response.

## 4 Discussion

In this preliminary study, we have shown for the first time that CW NIR laser has the capability to augment early antibody responses after ID vaccination with the inactivated vaccine without inducing a potentially harmful allergenic IgE response. Contrary, the standard alum adjuvant induced significantly delayed antibody responses, and both licensed chemical adjuvants of alum and MF59 induced IgE responses to the ID vaccination. These findings would significantly contribute to the design of candidate vaccine formulations by offering a safe and effective option of adjuvant for better safety and efficacy in ID vaccination.

Out of the effort to combat the recent coronavirus disease 2019 (COVID-19) pandemic, the development of effective vaccine is considered to be a sole medical measure to stop further community transmissions of the highly transmissible virus and ultimately end the pandemic in the absence of an efficacious therapeutic [68–71]. In a short period of time, while waiting for the development and approval of several vaccine candidates, the COVID-19 pandemic already posed unprecedented health and economic damages to our society [72]. Therefore, an approach to support rapid vaccine development in preparation for the future pandemic is highly desired to reduce mortality and morbidity caused by emerging pandemic threats. The use of chemical or biological adjuvants is a standard approach to confer protection with modern inactivated vaccines which often show insufficient efficacy [20]. However, only a limited number of adjuvants have been used in clinically approved vaccines due to their undesired local or systemic toxicity [27, 28]. Since prophylactic vaccines need to be broadly administered to healthy subjects, the use of chemicals or biologicals with potential side effects is not desirable in the initial phase of the pandemic when uninfected healthy populations need to be protected in a timely manner. The laser is a physical parameter and with the correct range poses no risk of side effects [31–33]. In order to promptly establish herd immunity with vaccination, the laser-based adjuvant, which safely augments early antibody responses to the vaccine, would be a feasible option for the candidate vaccines in the initial phase of the pandemic.

Although the use of NIR wavelengths as compared to visual wavelengths pushes effective limit up to a depth of a few millimeters, laser adjuvant is not compatible with most of the modern vaccines, which are administered via the standard intramuscular or subcutaneous routes [31, 32], posing unique technical challenges to this modality. Interestingly, laser adjuvant has been shown to augment humoral and antigen-specific CD4^+^ and CD8^+^ T cell responses to intramuscular immunization in a mouse model using a hair-like optical fiber [73], suggesting that laser adjuvant has the potential to augment the immune response to intramuscular vaccines. This possibility has not been well explored to date. The future study on this application would involve a preclinical proof-of-principle study as well as the development of an appropriate optical device.

At this time, it is unclear why the alum adjuvant produces a slower response compared to the NIR laser adjuvant. Since alum adjuvant is reported to induce tissue damage in host cells [74], it is possible that it damages or diminishes the function of migDCs *in situ* in the skin, which are critical for antibody responses to ID vaccination. In our previous study, the CC-chemokine receptor (CCR7^−/−^) mice, which lack CCR7-dependent migration of migDCs in the skin, failed to mount significant antibody responses to the NIR laser adjuvant in the context of ID vaccination [36], which indicates an indispensable role of migDCs in this system. Contrary, laser adjuvant is mostly regarded as nontissue damaging [31–33]. There is evidence of diverse effects of exposures with low-power NIR light, which are broadly defined as photobiomodulation (PBM) [75]. PBM is featured with specific activation of mitochondrial retrograde signaling including reactive oxygen species, cyclic AMP, nitric oxide (NO), and intracellular calcium via mitochondrial cytochrome c oxidase [75, 76], activation of ion channels including transient receptor potential channels [77], modulation of enzymes including mitogen-activated protein kinase, extracellular signal-regulated kinase, and protein kinase B [78], and modulation of effector molecules including growth factors [79, 80], inflammatory cytokines [81], and heat shock proteins [77], leading to activation of transcription factors and broad biological effects including cell migration and proliferation, cell differentiation, suppression of inflammation, decrease in apoptosis, and alleviation of oxidative stress [77, 82–84]. Likewise, laser adjuvant has been validated to modulate skin-resident migDCs without inducing overt inflammation in mice [36, 37] and humans [38]. Thus, because of its noninvasive nature, laser adjuvant would readily augment the immune response in the context of ID vaccination via activation of migDCs.

Although it has been shown that the effect of the NIR laser adjuvant is mediated by CD103^+^ migDCs, it did not induce a T_H_1-skewed response. In particular, in our previous study, the NIR laser has been shown to induce migration of Langerin^+^ (Lang^+^) and CD11b^−^Lang^−^ migDC subsets and recruit CD11b^+^Ly6C^+^ monocytes [36]. Since depletion of Lang^+^ cells, which include CD103^+^ DCs [63–65], abolished the effect of the NIR laser on the migration of DCs and antibody responses to ID vaccination [36], the effect of the NIR laser adjuvant is considered to be mediated by coordination between Lang^+^ and other DC subsets. In addition, since CCR2^−/−^ mice, which are used to test the contribution of inflammatory monocytes and monocyte-derived DCs [85], induced the heightened T_H_2 response in this study [36], coordination of these critical DC subsets in the skin likely mediates the adjuvant effect of the NIR laser and induction of a mixed T_H_1–T_H_2 response with the NIR laser-adjuvanted vaccine.

Since NIR laser adjuvant has the ability to safely induce selective signaling and activation of the cross-presenting CD103^+^ DC subpopulation [36, 37], which is known to prime cytotoxic T cells against tumors [86, 87] and orchestrate trafficking of effector T cells into the tumor microenvironment [88], it holds a potential to be broadly combined with cancer therapy to augment the anti-cancer immune response. In addition, NIR laser adjuvant is free from potentially harmful drug–drug interactions and can be broadly combined with current and candidate chemical or biological agents. NIR laser could therefore represent a paradigm-shifting approach to use “light” as a drug for cancer treatment. Currently, such cancer therapy is not available, and this laser-based technology could be considered as a potential candidate for a new class of cancer immunotherapy.

The NIR laser adjuvant opens a major pathway toward important goals including the elimination of chemical adjuvant and the development of needleless vaccination. In response to this, future work will include the development of low-cost and easy-to-use portable devices and human clinical studies to evaluate the safety and efficacy of the NIR laser adjuvant. NIR laser has been used in the field of medicine for more than three decades, and there are multiple U.S. Food and Drug Association- and European Medicines Agency-approved laser devices, which would be a significant advantage to advance to the clinical study.

With the proven safety and efficacy, ultrashort pulsed and fractional laser made clinical stages [31]. Since the first demonstration of the effect in cancer patients in Russia, the use of ultrashort pulsed laser expanded to ID influenza and hepatitis B vaccines in human subjects with positive results [31–33, 89]. On the other hand, a group at the Medical University of Vienna sponsored by Pantec Biosolutions AG successfully applied the fractional laser to ID administration of seasonal influenza vaccine using the P.L.E.A.S.E.^®^ system and completed phase I clinical trial (NCT02988739). However, the recent withdrawal of the ID influenza vaccine by Sanofi Pasteur from the US market will likely negatively impact the clinical translation of this technology.

In summary, our results demonstrated that CW NIR laser possesses a unique ability to augment early antibody responses to ID vaccination via CD103^+^ DCs in the skin. In addition, unlike the licensed chemical adjuvants, the NIR laser adjuvant produces a mixed T_H_1–T_H_2 response without inducing an allergenic IgE response to ID vaccination. These findings would contribute to the design of candidate vaccines by offering a safe and effective laser-based adjuvant to support accelerated vaccine development and timely combat emerging infectious diseases.

## List of nonstandard abbreviations

AC: Achromatic
Akt: Protein kinase B
Batf3: The basic leucine zipper transcription factor ATF-like 3
CCR: The CC-chemokine receptor
CD: Cluster of differentiation
COVID-19: Coronavirus disease 2019
CW: Continuous wave
COX: Cytochrome c oxidase
DC: Dendritic cell
ELISA: Enzyme-linked immunosorbent assay
EMA: European Medicines Agency
FDA: U.S. Food and Drug Association
HSPs: heat shock proteins
ID: Intradermal
IgG/E: Immunoglobulin G/E
LN: Lymph node
MAPK: Mitogen-activated protein kinase
migDCs: Migratory DCs
MGH: Massachusetts General Hospital
NA: Numerical aperture
NIR: Near-infrared
Nd:YAG: Neodymium-doped yttrium aluminum garnet
NO: Nitric oxide
PBM: Photobiomodulation
PC: Plano-convex
ROS: Reactive oxygen species
TMB: Tetramethylbenzidine
TME: Tumor microenvironment
T_H_1/2: T helper cell type 1/2
TRP: Transient receptor potential
WT: Wild type
XCR1: X-C motif chemokine receptor 1
